# Clinical efficacy of IgM-enriched immunoglobulin as adjunctive therapy in neonatal and pediatric sepsis: a systematic review and meta-analysis

**DOI:** 10.3389/fped.2023.1239014

**Published:** 2023-08-11

**Authors:** Ener Cagri Dinleyici, Georg Frey, Ermira Kola, Ulrike Wippermann, Artur Bauhofer, Alexander Staus, Peter Griffiths, Muhamad Azharry, Rinawati Rohsiswatmo

**Affiliations:** ^1^ Department of Pediatrics, Eskisehir Osmangazi University Faculty of Medicine, Eskisehir, Türkiye; ^2^ Klinik für Neonatologie, Darmstädter Kinderkliniken Prinzessin Margaret, Perinatalzentrum Südhessen, Darmstadt, Germany; ^3^ Pediatric Intensive Care Unit, University Hospital Center “Mother Teresa”, Tirana, Albania; ^4^ Corporate Medical Affairs, Biotest AG, Dreieich, Germany; ^5^ Corporate Clinical Research & Development, Biotest AG, Dreieich, Germany; ^6^ Medical and Scientific Affairs, Biotest UK, Birmingham, United Kingdom; ^7^ Department of Child Health, Neonatology Division, Dr. Cipto Mangunkusumo National Central General Hospital, Jakarta, Indonesia

**Keywords:** IgM-enriched immunoglobulin, neonate, pediatric, sepsis, mortality, infection, meta-analysis

## Abstract

**Background:**

Sepsis is a major cause of mortality and morbidity globally, with around one-quarter of all sepsis-related deaths occurring in children under the age of 5. We conducted a meta-analysis and systematic review of the literature to evaluate the clinical effectiveness of an IgM-enriched immunoglobulin preparation in pediatrics patients and neonates with sepsis.

**Methods:**

Systematic searches of PubMed, the Cochrane Library and Embase databases were performed in November 2022, with no date limitations, to identify studies in which IgM-enriched immunoglobulin was used as adjunctive therapy in neonatal and pediatric patients with sepsis.

**Results:**

In total, 15 studies fulfilled the eligibility criteria, 13 neonatal studies and 2 pediatric studies. Pooled estimates from all studies indicated that mortality rates were significantly lower in patients who received treatment with the IgM-enriched immunoglobulin compared with controls (OR 0.41; 95% CI 0.32–0.55). Further analyses in neonatal studies, alone, showed a significant benefit with longer treatment durations (>3 days) vs. the recommended treatment duration (3 days) (OR 0.32; 95% CI 0.22–0.47) vs. (OR 0.61; 95% CI 0.41–0.92). Treatment with IgM-enriched immunoglobulin was associated with a lower mortality risk compared with controls in prospective studies vs. retrospective analyses (OR 0.37; 95% CI 0.27–0.51) vs. (OR 0.73; 95% CI 0.41–1.30).

**Conclusions:**

This systematic review suggests that adjunctive treatment with IgM-enriched immunoglobulin may reduce the risk of mortality in neonatal and pediatric populations. However, large randomized controlled trials are required to further substantiate and evaluate these findings.

## 1. Introduction

Sepsis is a life-threatening organ dysfunction caused by a dysregulated host response to infection (1, 2). In the pediatric Surviving Sepsis Campaign International Guidelines, which apply to all patients from 37 weeks gestational age at birth to 18 years old, septic shock is defined as severe infection leading to cardiovascular dysfunction (including hypotension, need for treatment with a vasoactive medication, or impaired perfusion). “Sepsis-associated organ dysfunction” is defined as severe infection leading to cardiovascular and/or non-cardiovascular organ dysfunction (3, 4). A consensus definition of neonatal sepsis is still a matter of debate (5).

Approximately one-half of pediatric patients with sepsis have underlying disease such as chronic lung disease, congenital heart disease or neuromuscular disease (6, 7), with sepsis most commonly the result of diarrheal disease or lower respiratory infections (8). However, community-acquired meningococcal infection can cause sepsis even in healthy children.

Neonates, in particular preterm neonates, are at a higher risk of infection as a result of the functional immaturity of their immune system (9–11). Immunoglobulin (Ig) G is transferred from mother to fetus, but the majority isn’t acquired until the last month of pregnancy (10). IgM and IgA do not cross the placenta (12). Consequently, preterm or low birth weight (LBW) neonates are hypogammaglobulinemic (12) in addition to having an immature adaptive immune system and, as a result, are at greater risk of severe infection and mortality (13).

Despite advances in treatment, sepsis is still a significant cause of morbidity and mortality, particularly in neonates and pediatric populations, and even more so in low-and-middle-income countries (9, 13). In 2017, there were an estimated 48.9 million cases of sepsis globally, of which 42% were in children aged under 5 years (8). For those who survive, sepsis is associated with long-term comorbidities (14, 15), including poor neurodevelopmental and growth outcomes in early childhood in preterm and LBW neonates (16, 17) and at least moderate disability in almost one-fifth of older children (15).

Intravenous immunoglobulin (IVIG) preparations, that primarily contain IgG, have been suggested to have benefits as adjunctive therapy in patients with sepsis. However, evaluation of polyclonal IVIG adjunctive therapy in neonates showed no survival benefit in the large multinational International Neonatal Immunotherapy Study (INIS) (18). An alternative immunoglobulin preparation (see methods) enriched with IgM and IgA in addition to IgG, denoted IgM-enriched immunoglobulin for this review, is available for the treatment of sepsis. Said IgM-enriched immunoglobulin is considered to offer greater antibacterial and immunomodulatory activity (19–21), with a higher affinity for the lipopolysaccharides of gram-negative bacteria compared with IgG (19, 22), and the capacity to neutralize bacterial toxins (23) compared with IVIG. In adults, treatment with IgM-enriched immunoglobulin has been associated with improved survival (24–29), however data in neonatal and in particular pediatric populations are limited. Nevertheless, given the potential benefits of IgM-enriched immunoglobulin, in the context of the neonate's compromised immunity, it would be reasonable to believe that IgM-enriched immunoglobulin may have a beneficial effect in neonates.

Systematic meta-analyses have conducted subgroup analyses on adjunctive therapy with IgM-enriched immunoglobulin in neonatal patient populations (29–31). However, the limited studies have precluded robust conclusions on its therapeutic potential and emphasize the need for larger trials. The recent publication of further studies evaluating adjunctive treatment with IgM-enriched immunoglobulin in pediatric and neonatal sepsis, have prompted this systematic review, the aim of which is to provide an updated analysis and review of studies that assess the effectiveness of IgM-enriched immunoglobulin as adjunctive therapy in pediatric and neonatal sepsis.

## 2. Methods

A systematic review and meta-analysis were performed using the Preferred Reporting Items for Systematic Review and Meta-Analyses (PRISMA) 2020 guidelines.

### 2.1. Data sources

A systematic search of PubMed, Cochrane Library, and Embase databases was conducted in November 2022 and was not date limited. The search strategy consisted of ((“IgM-enriched immunoglobulin”) or (“IgM enriched immunoglobulin”) or (“IgM and IgA-enriched immunoglobulin”) or (“IgM/IgA enriched immunoglobulin”) or (IVIGMA) or (IgGAM) or (“IgMA enriched IVIG”) or (“IgMA-enriched IVIG”) or (“polyvalent immunoglobulins”) or (“immunoglobulin M preparation”) or (“immunoglobulin preparation containing IgG, IgM and IgA”) or (pentaglobin or pentaglobulin)) and (sepsis or septic shock). Additional studies were identified by reviewing the reference lists of relevant articles and a manual search of the internet. In addition, results of an unpublished study (32) were included in the meta-analysis.

### 2.2. Eligibility criteria

Two authors (UW and AB) evaluated the studies independently to determine their eligibility for inclusion in the meta-analysis. Trials were included if they: (1) compared IgM-enriched immunoglobulin, specifically Pentaglobin® Biotest Germany (12% IgM, 12% IgA and 76% IgG) and standard therapy (standard therapy only or placebo and standard therapy); (2) enrolled neonatal or pediatric patients with sepsis; and (3) provided mortality data. The primary outcome was all-cause mortality. Studies on prophylactic use of IgM-enriched immunoglobulin or studies where normal IVIG was used were excluded. Any disagreements between authors were resolved by consensus.

### 2.3. Data extraction

Two authors (UW and AB) extracted the data independently from each eligible study, including the lead author, year of publication, study design, number of patients, age for pediatric patients and gestational age and birthweight for neonatal patients, dose and duration of treatment, and definition of mortality. The number of patients in the IgM-enriched immunoglobulin treatment and control groups in each study were recorded as well as the number of deceased patients.

### 2.4. Quality assessment

The methodological quality of the studies included in our analysis was assessed independently by two authors (UW and AB). Randomized controlled studies (RCTs) were assessed using the Cochrane Collaboration's tool for assessing risk of bias (33). Each study was examined for selection bias, performance bias, attrition bias, detection bias and reporting bias, with each criterion graded as either low, high or unclear risk. Observational studies were assessed using the Newcastle–Ottawa Scale (34) which examined selection bias, comparability and outcome. Each study was awarded up to nine stars, with those regarded as high-quality studies receiving ≥6 stars.

### 2.5. Statistical analyses

All statistical analyses were performed with R Version 4.1.1. Heterogeneity was evaluated using chi–squared tests and the *I*^2^ index. If the *I*^2^ index was between 50% and 75%, heterogeneity was considered moderate. If the *I*^2^ index was >75%, heterogeneity was evaluated as considerable. Potential publication bias was assessed using funnel plots, with asymmetry evaluated using the Egger's test. Independent of heterogeneity a conservative approach was applied by using a Mantel–Haenszel random effects model. A value of 0.5 was added to cells where the mortality count was zero.

A subgroup analysis was performed to evaluate whether any mortality benefit associated with treatment with IgM-enriched immunoglobulin was affected by pediatric vs. neonatal populations. All subsequent subanalyses were performed on studies in neonates only, with further analyses on the effects of: recommended IgM-enriched immunoglobulin treatment dose according to the summary of product characteristics (SmPC) (35) (Pentaglobin® Biotest Germany, 250 mg/kg/day for 3 consecutive days) vs. a greater number of days administered (250 mg/kg/day for >3 consecutive days); patients with proven vs. suspected sepsis; retrospective vs. prospective studies; and studies published before 2000 vs. studies published after 2000 (all studies that were published pre 2000 were conducted and completed before the year 2000 and those published post 2000 were conducted after the year 2000).

## 3. Results

### 3.1. Identification of studies

**Figure 1:**
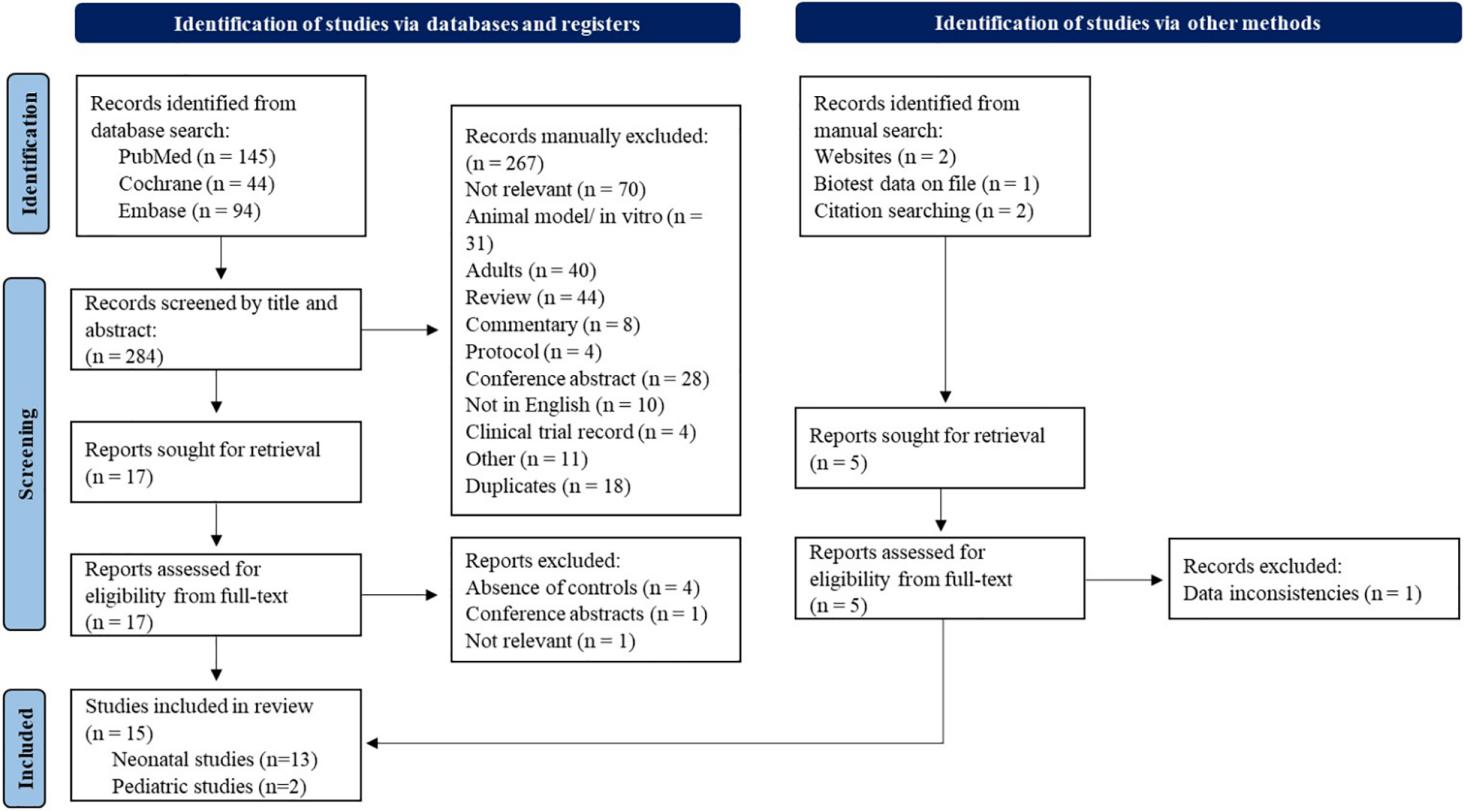
Flowchart of the study selection process.

The flow chart of the study selection procedure is shown in Figure 1. The initial search identified 145 studies in PubMed, 44 in the Cochrane Library and 94 from Embase. In addition, four studies were identified by citation searching and manual search. An unpublished report was also identified for inclusion (32). The titles and abstracts of all studies found in the search were screened and 22 articles were evaluated for eligibility from their full-text manuscript. In total, 15 studies (32, 36–49) fulfilled the eligibility criteria (two pediatric and 13 neonatal studies) and were included in the final meta-analysis. These studies included a total of 1,727 patients (1,549 neonates and 178 pediatric patients).

### 3.2. Neonate study details

The details of the 13 neonatal studies included in the meta-analysis are shown in Table 1. Of these, nine were controlled studies, eight of which were randomized (32, 36–38, 43, 46, 48, 49) and one was non-randomized (47). The remaining four studies were retrospective, controlled data analyses (41, 42, 44, 45). The studies included varied in size from 28 to 496 patients. The mean gestational age was reported for nine of the neonatal studies and varied from 26 to 35 weeks. Two of the remaining studies defined how many neonates were preterm (<37 weeks) (44, 47), one defined the median gestational age (46) and the final study did not report gestation (36). The mean birthweight was reported for the same nine studies as those with mean gestational age and varied from 0.7–2.1 kg. For the four remaining studies, three studies reported the percent of patients with LBW (defined as <2.5 kg) (36, 44, 47) and one the median birthweight (46). Dose of IgM-enriched immunoglobulin preparation was the same for all neonatal studies (250 mg/kg/day), however, four studies extended the duration of treatment beyond the recommended 3 days, with durations of 4 days (36–38) and 5 days (43). Definitions of mortality varied between the studies included in the analyses: mortality was not defined for five studies (32, 36–38, 48); mortality was defined as deceased or discharged for six studies (41–43, 45–47); and mortality was defined as short-term mortality at 7 and 21 days (49) and 7 and 28 days (44) for the remaining two studies.

**Table 1:** Characteristics of the 13 controlled neonatal studies included in the review.

First author, city, country	Study design^¶¶^	Size of study population (*N*)	Gestation (weeks), mean (SD)*, control group/IgM-enriched immunoglobulin group	Birthweight (kg), mean (SD)*, control group/IgM-enriched immunoglobulin group	IgM-enriched immunoglobulin treatment duration (250 mg/kg/day)	Comparator (SoC or placebo)***	Primary endpoint(s)
Haque (37) Riyadh, Saudi Arabia	Randomized	60	35 (28–37)/33 (28–37)^†^	1.5 (0.9–1.7)/1.3 (0.9–1.6)^†^	4 days	5 ml/kg/day 10% dextrose	Mortality^¶^
Erdem (48) Ankara, Turkey	Randomized	44	35 ± 2/34 ± 2	2.1 ± 0.4/2.1 ± 0.4	3 days	SoC	Mortality^¶^
Haque (38) Riyadh, Saudi Arabia	Randomized	119^§§^	30 (24–42)/30 (24–42)^†^	1.0 (0.5–4.2)/1.0 (0.5–4.2)^†^	4 days	SoC	Mortality^¶^
Samatha (47) Bangalore, India	Randomized	60	60%/87% preterm	80%/90% LBW^‡^	3 days	SoC	Mortality**
Hellwege (32) Hamburg, Germany	Randomized	88	32 ± 4/32 ± 5	1.7 ± 1.0/1.7 ± 1.0	3 days	5 ml/kg 5% low salt human albumin	Mortality (secondary endpoint)^¶^
Capasso (41) Naples, Italy	Retrospective, data collection	79	28 ± 4/27 ± 3	0.9 ± 0.3/1.0 ± 0.4	3 days	SoC	Mortality between 7 and 21 days; total mortality** (secondary endpoint)^††^
Akdag (46) Ankara, Turkey	Randomized	102^||||^	31 (25–40)/30 (24–41)^§^	1.4 (0.6–4.3)/1.3 (0.6–3.9)^§^	3 days	5 ml/kg of normal saline/day	Mortality**
Abbasoğlu (44) Ankara, Turkey (45)	Retrospective, data collection	63	31 ± 5/30 ± 4	1.7 ± 0.8/1.4 ± 0.7	3 days	SoC	Mortality**
Pal (36) Kolkata, India	Randomized	496	NR	84%/81% LBW^‡^	4 days	SoC	Mortality^¶^
Jindal (43) Bhavnagar, India	Randomized	60	31 ± 2/31 ± 2	1.2 ± 0.2/1.3 ± 0.1	5 days	Soc	Mortality** discharge rate
Boonsopa (44) Phitsanulok, Thailand	Retrospective, data collection	28	71%/86% preterm	50%/79% LBW^‡^	3 days	SoC	Mortality at Day 7 and 28^‡‡^
Capasso (42) Naples, Italy	Retrospective, data collection	78	26 ± 2/26 ± 2	0.7 ± 0.1/0.7 ± 0.2	3 days	SoC	Mortality between 7 and 21 days; total mortality** (secondary endpoint)^††^
Nassir (49) Baghdad, Iraq	Randomized	272	30 ± 3/30 ± 3	1.3 ± 0.1/1.3 ± 0.1	3 days	SoC	Mortality between 7 and 21 days; length of hospitalization

IVIG, IV immunoglobulin; LBW, low birth weight; NR, not reported; SD, standard deviation; SoC, standard of care.

* Mean (SD) unless defined otherwise.

^†^ Mean (range).

^‡^ LBW not defined.

^§^ Median (range).

^||^ LBW defined as <2.5 kg.

^¶^ Mortality was not defined.

** Mortality rate: deceased or discharged (where mortality is not explicitly defined but length of hospitalization has been reported, mortality is presumed to be deceased or discharge).

^††^ Total mortality was secondary endpoint and is used in this meta-analysis.

^‡‡^ Mortality at day 28 was used in this meta-analysis.

^§§^ Study also included IVIG treatment arm which was excluded from total study population; matched control patients were selected randomly from babies treated in same period.

^||||^ Study also included pentoxifylline ± IgM-enriched immunoglobulin treatment arms which were excluded from total study population.

^¶¶^ Study design was analysed in detail in Supplementary Figures S1–S3.

*** Standard of care is according to local sepsis guidelines.

### 3.3. Pediatric study details

The details of the two pediatric studies included in the meta-analysis are shown in Table 2. Both were RCTs (39, 40). A total of 78 and 100 patients were included in each study, with a mean age from 2.4 months (39) to 2.1 years (40). Dose of IgM-enriched immunoglobulin preparation was different in these studies; the recommended dose (250 mg/kg/day) was used in one study (40) and a higher dose (400 mg/kg/day) for the remaining study (39), with the same duration (3 days) for both. Definitions of mortality were deceased or discharged for one study (39) and mortality in ICU for the other (40).

**Table 2:** Characteristics of two pediatric randomized studies included in the review.

First author, City, Country	Study design	Study population (*N*)	Age control group/IgM-enriched immunoglobulin group	IgM-enriched immunoglobulin dose and duration	Comparator (SoC or placebo)^||^	Primary endpoint
El-Nawawy (39) Alexandria, Egypt	Randomized	100	2.4 (1.0–24.0)/3.8 (1.0–24.0) months*	400 mg/kg/day for 3 days	SoC	Mortality^‡^
Kola (40) Tirana, Albania	Randomized	78	1.8 (0.9–2.7) 2.1 (1.1–3.1) years^†^	250 mg/kg/day for 3 days	SoC	Mortality in ICU^‡^

ICU, intensive care unit; SD, standard deviation; SoC, standard of care.

* Mean (min, max).

^†^ Mean (95% confidence interval).

^‡^ Mortality rate: deceased or discharged.

^||^ Standard of care is according to local sepsis guidelines.

### 3.4. Methodological quality of included RCTs

The results of the Cochrane Collaboration's risk of bias assessment of RCTs are reported in Supplementary Figures S1 and S2.

#### 3.4.1. Allocation

The method of random sequence generation was described for four studies (32, 40, 46, 47) but was undefined for a further six studies (36, 37, 39, 43, 48, 49). The blinding of randomization to conceal the allocation sequence was described for three studies (37, 40, 46) however for seven studies there was insufficient information to determine whether allocation had been adequately concealed (32, 36, 39, 43, 47–49).

#### 3.4.2. Blinding

Only two studies reported blinding of participants, personnel and/or outcome (37, 46) with no reports of blinding for the remaining nine studies (32, 36, 38–40, 43, 47–49). However, it should be noted, that it is unlikely that bias due to lack of blinding would have had an impact on mortality outcome (33).

#### 3.4.3. Incomplete outcome data, selective reporting and other bias

Only one study had incomplete outcome data because of missing data following premature termination and dropout, and other bias potentially affecting inferential statistical analysis (32). The remaining ten studies (36–40, 43, 46–49) had no missing mortality data, and no other identifiable potential sources of bias. Conversely, it was not possible to ascertain if there was any selective reporting for these ten studies (36–40, 43, 46–49), but the absence was clear for the study by Hellwege et al. (32).

### 3.5. Newcastle–Ottawa quality assessment

The result of the quality assessment of the observational studies is reported in Supplementary Table S3. Two studies (41, 42) scored 6 stars and were regarded as high-quality studies and the remaining two studies (44, 45) scored <6 stars.

### 3.6. Primary outcomes

**Figure 2:**
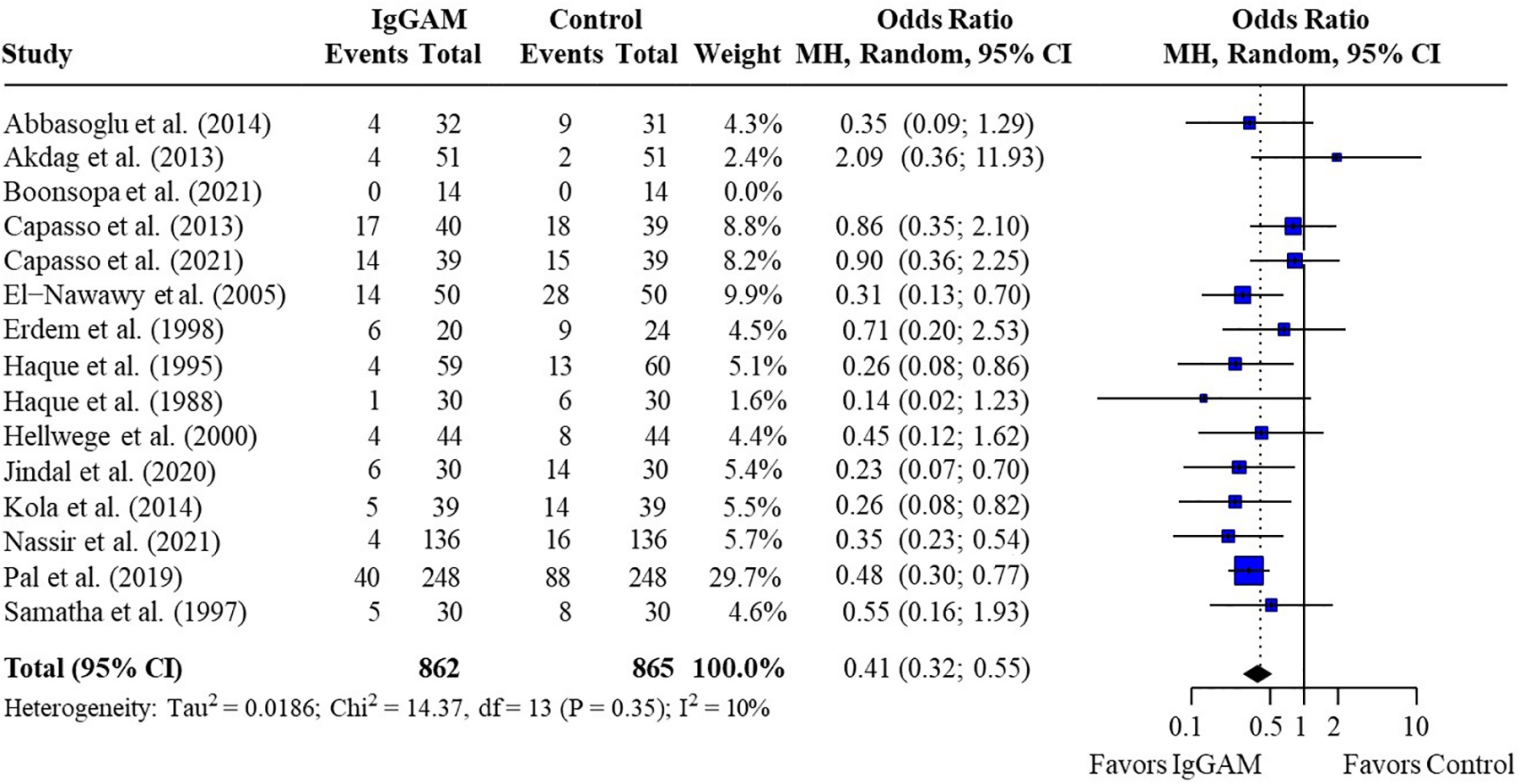
Effect of IgM-enriched immunoglobulin treatment on mortality rates in all patients with sepsis (pediatrics and neonates). CI, confidence interval; IgGAM, IgM-enriched immunoglobulin; MH, Mantel–Haenszel.

Pooled estimates from all neonatal and pediatric studies indicated that mortality rates were significantly lower in patients who were treated with IgM-enriched immunoglobulin compared with their respective controls [odds ratio (OR) 0.41; 95% confidence interval (CI) 0.32–0.55] (Figure 2). Statistical homogeneity was met (*I*^2^ = 10%).

**Figure 3:**
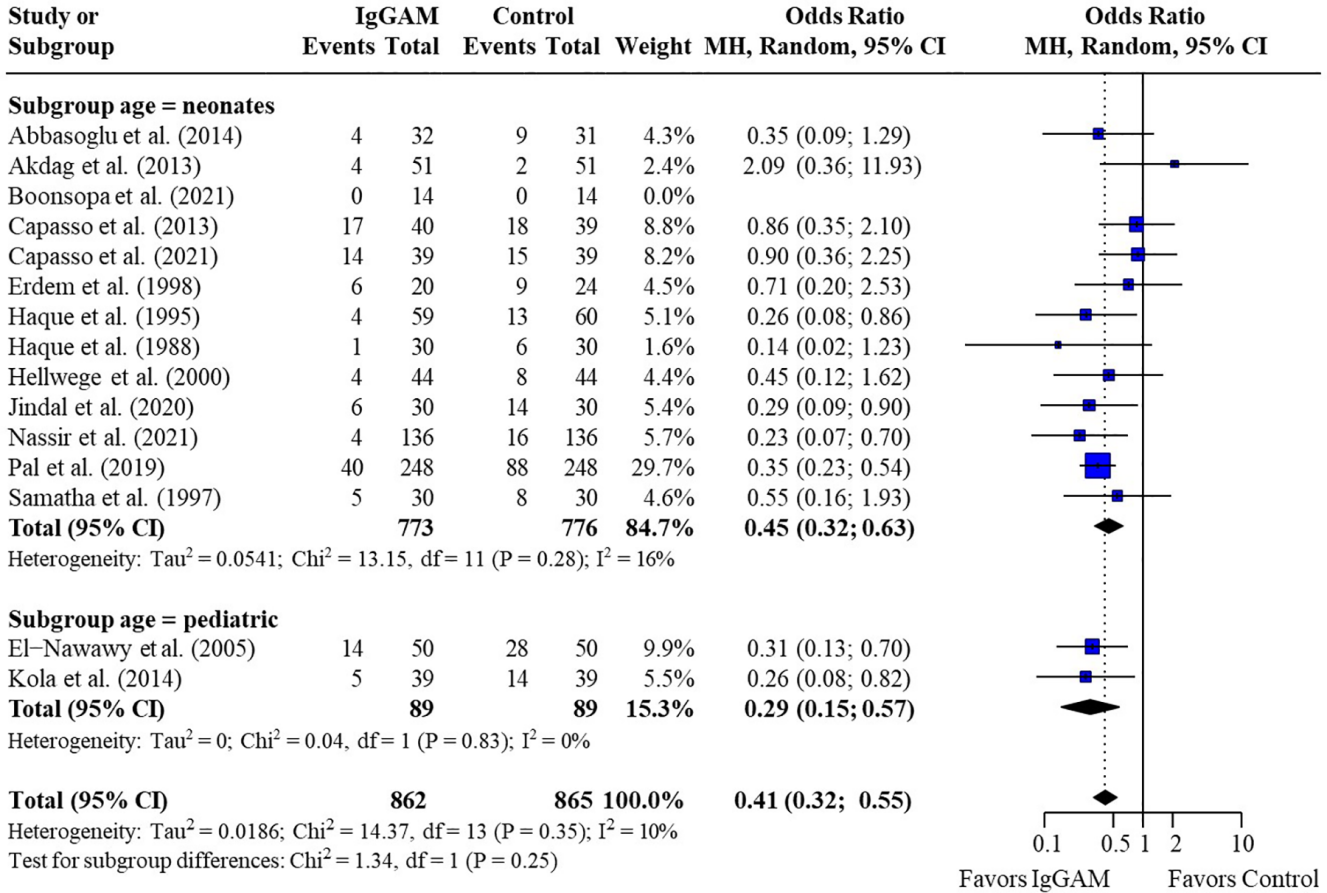
Subgroup analyses to compare the effect of IgM-enriched immunoglobulin treatment on mortality rates in neonatal and pediatric sepsis. CI, confidence interval; IgGAM, IgM-enriched immunoglobulin; MH, Mantel–Haenszel.

A subgroup analysis was performed to compare mortality benefit in neonatal studies and pediatric studies; mortality rates remained significantly lower in patients treated with IgM-enriched immunoglobulin compared with controls for both neonatal and pediatric populations (OR 0.45; 95% CI 0.32–0.63) vs. (OR 0.29; 95% CI 0.15–0.57), respectively, with no significant differences observed between populations (*p* = 0.25) (Figure 3 and Table 3). All other analyses were based on neonatal studies only.

**Table 3:** Results of subgroup analysis based on different modifiers.

	K	N	OR (95% CI)	*P*	Study heterogeneity			P (Between-group comparison)
					Chi	*I*^2^ (%)	*P*	
Patients								
Neonates	13	1,549	0.45 (0.32; 0.63)	<0.001	13.15	16	0.28	0.25
Pediatrics	2	178	0.29 (0.15; 0.57)	<0.001	0.04	0	0.83	
Treatment duration*								
Recommended (3 days)	9	814	0.61 (0.41; 0.92)	0.019	7.14	2	0.41	0.02
Longer (>3 days)	4	735	0.32 (0.22; 0.47)	<0.001	0.87	0	0.83	
Sepsis diagnosis^†^								
Proven	10	1,116	0.40 (0.27; 0.60)	<0.001	10.11	11	0.34	0.55
Suspected	5	215	0.51 (0.25; 1.05)	0.067	1.62	0	0.80	
Study design								
Retrospective	4	248	0.73 (0.41; 1.30)	0.292	1.55	0	0.46	0.04
Prospective	9	1,301	0.37 (0.27; 0.51)	<0.001	7.38	0	0.50	
Publication year								
New studies (>2000)	8	371	0.48 (0.30; 0.77)	0.003	10.67	44	0.10	0.7
Old studies (<2000)	5	1,178	0.42 (0.23; 0.76)	0.004	2.46	0	0.65	

Chi, Pearson's Chi–squared test statistic; *I*^2^, heterogeneity statistic; K, number of studies; N, number of patients; OR, odds ratio; P, *p*-value; CI, confidence interval.

* Recommended treatment is (250 mg/kg/day for 3 days) (34) compared with longer treatment duration >3 days.

^†^ Studies that compared treatment effect on mortality in patients after microbiological confirmation of sepsis were included in both proven and suspected sepsis analyses (35, 36, 37, 46, 47).

**Figure 4:**
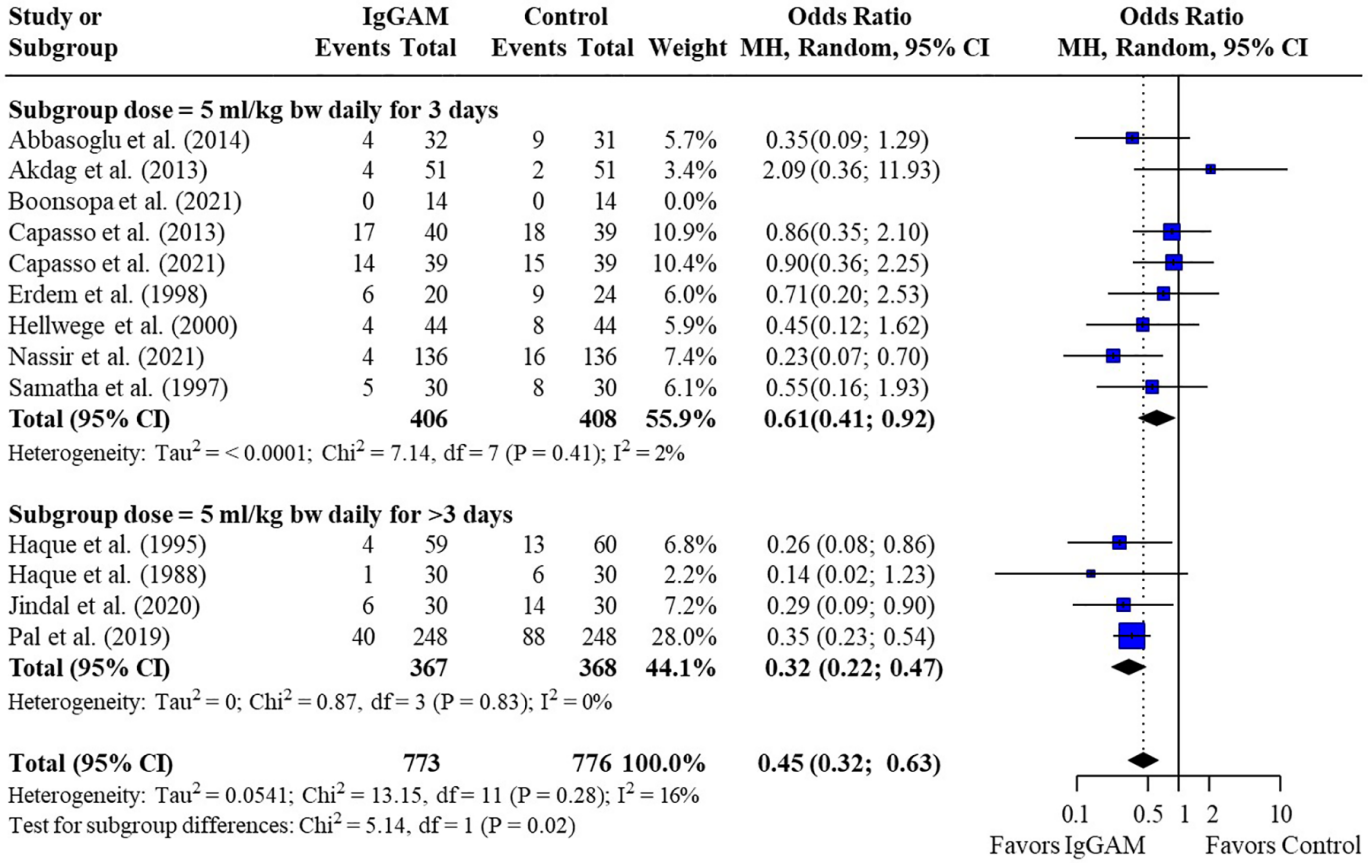
Subgroup analyses to compare the effect of a longer treatment duration (>3 days) of IgM-enriched immunoglobulin compared with the recommended regimen (3 days) on mortality rates in neonatal sepsis. BW, body weight; CI, confidence interval; IgGAM, IgM-enriched immunoglobulin; MH, Mantel–Haenszel.

**Figure 5:**
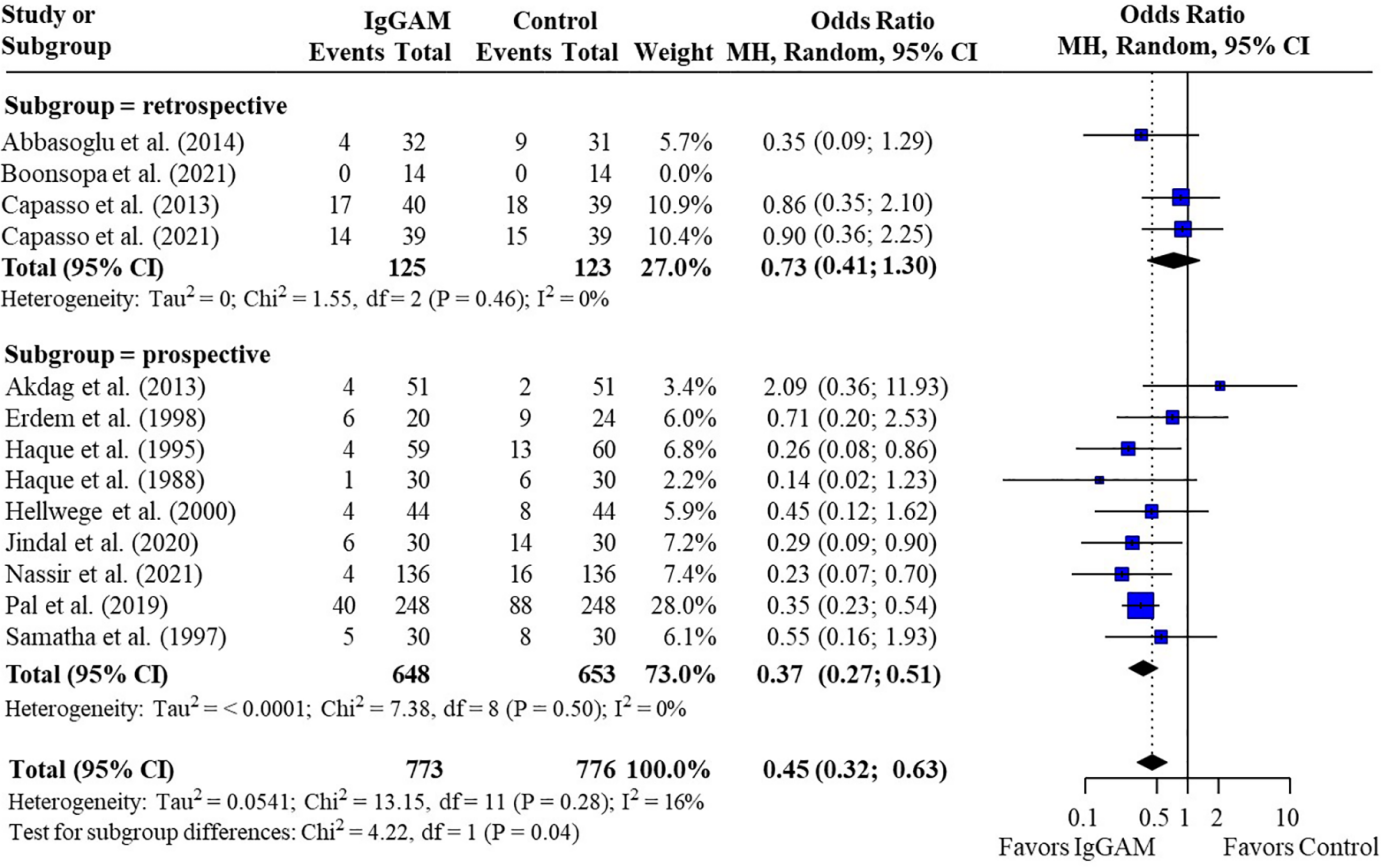
Subgroup analyses to compare the effect of IgM-enriched immunoglobulin treatment in retrospective vs. prospective studies on mortality rates in neonatal sepsis. CI, confidence interval; IgGAM, IgM-enriched immunoglobulin; MH, Mantel–Haenszel.

**Figure 6:**
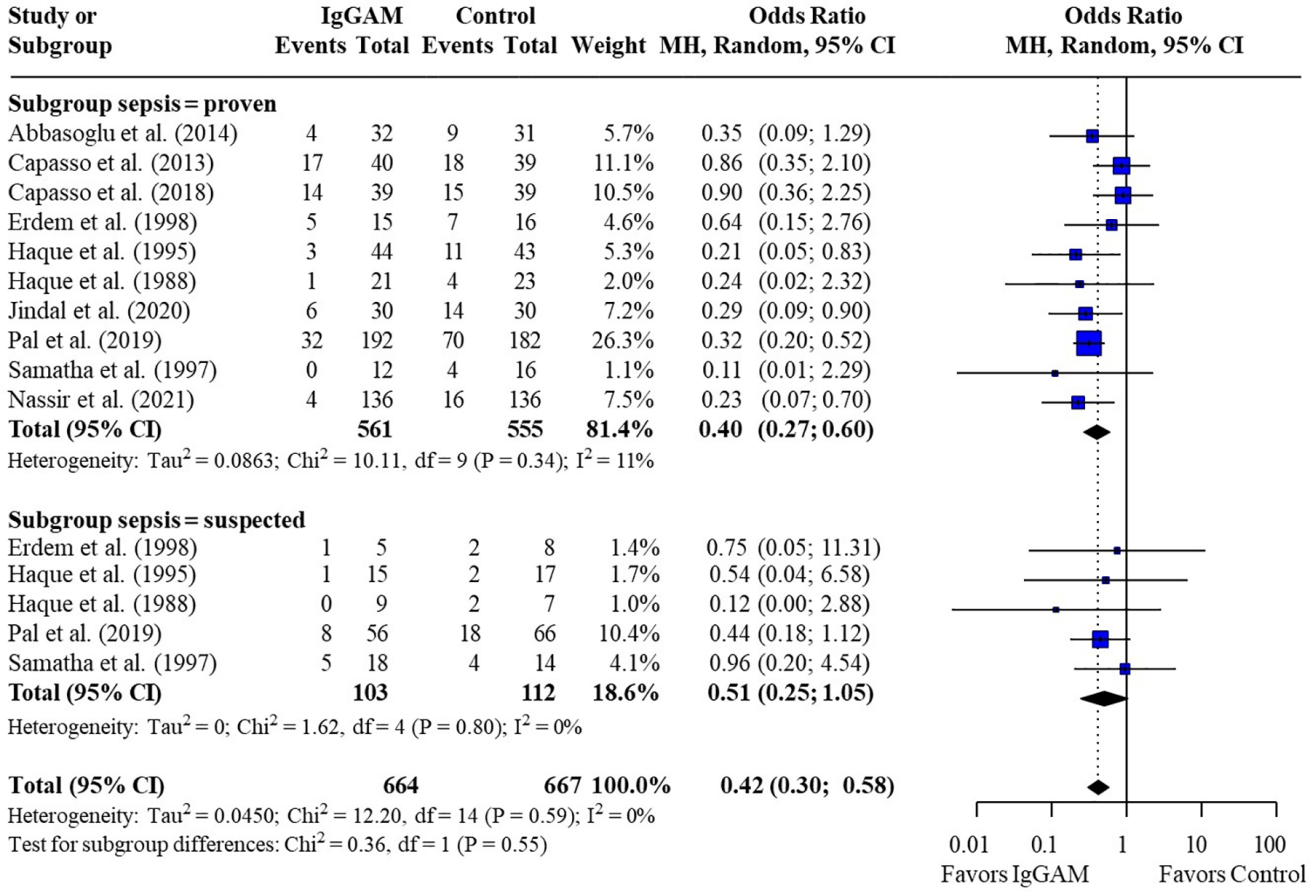
Subgroup analyses to compare the effect of IgM-enriched immunoglobulin treatment on mortality rates in neonates with proven or suspected sepsis. Studies which compared treatment effect on mortality in patients after microbiological confirmation of sepsis were included in both proven and suspected sepsis analyses (35, 36, 37, 46, 47). CI, confidence interval; IgGAM, IgM-enriched immunoglobulin; MH, Mantel–Haenszel.

**Figure 7:**
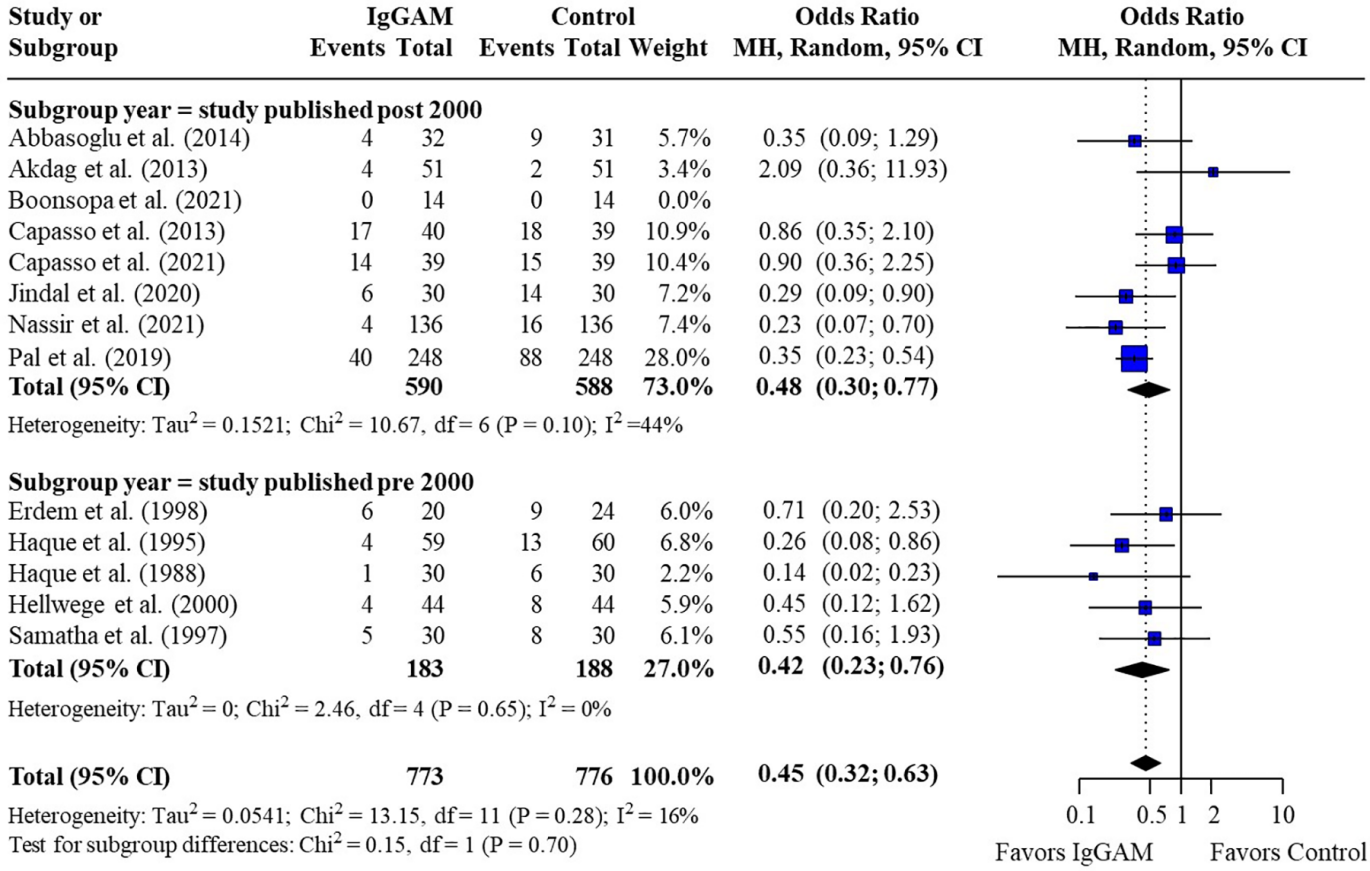
Subgroup analyses to compare the effect of IgM-enriched immunoglobulin treatment in older (pre 2000)* vs. newer (post 2000) studies on mortality rates in neonatal sepsis. *All studies pre and post 2000 were enrolled and published within these cut-off dates. CI, confidence interval; IgGAM, IgM-enriched immunoglobulin; MH, Mantel–Haenszel.

Subsequent subgroup analyses of neonatal studies observed that IgM-enriched immunoglobulin significantly reduced the mortality risk (*p* = 0.02) compared with controls according to the duration of treatment. Compared with controls, mortality risk was more markedly reduced with prolonged IgM-enriched immunoglobulin treatment (250 mg/kg/day for >3 days) (OR 0.32; 95% CI 0.22–0.47) vs. the recommended dose (250 mg/kg/day for 3 days) (OR 0.61; 95% CI 0.41–0.92) (Figure 4 and Table 3). A significant difference (*p* = 0.04) was also observed for the study design subgroup analysis, with a greater reduction in mortality risk compared with controls observed with IgM-enriched immunoglobulin treatment in prospective studies vs. the retrospective analyses (OR 0.37; 95% CI 0.27–0.51) vs. (OR 0.73; 95% CI 0.41–1.30) (Figure 5 and Table 3). There were no differences in comparative mortality risk for subgroup analyses for sepsis diagnosis (Figure 6 and Table 3) or publication year (Figure 7 and Table 3). Although treatment with IgM-enriched immunoglobulin was associated with a significantly lower risk of mortality compared with controls for patients with proven sepsis (*p* < 0.001), significance was not reached in the analysis of patients with suspected sepsis (*p* = 0.067).

### 3.7. Publication bias

**Figure 8:**
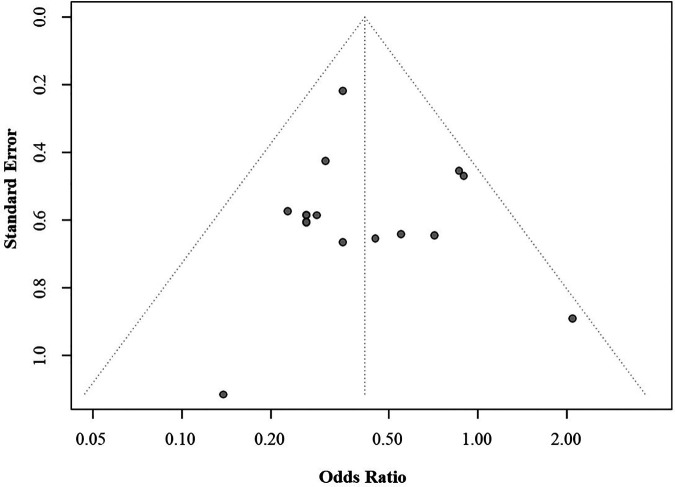
Funnel plot of mortality rates.

Assessment of publication bias showed that no bias was evident in the analyses, as indicated by the presence of all results within the funnel (Egger's test *p* > 0.05) (Figure 8).

## 4. Discussion

This meta-analysis evaluating IgM-enriched immunoglobulin as adjunctive therapy in neonatal and pediatric sepsis, included 13 studies in neonates (*n* = 1,549) and two studies in pediatric patients (*n* = 178). Overall, treatment with IgM-enriched immunoglobulin significantly reduced risk of mortality compared with controls in both populations.

The pathology and pathophysiology of sepsis differs between neonatal and pediatric patients. Neonates, especially preterm neonates, are highly susceptible to infection due to their immature immune system, which can lead to an inadequate or dysregulated immune response to pathogens (50, 51). In contrast, almost one-half of all pediatric patients who develop sepsis have a comorbid condition that increases their susceptibility to infection (52). The cause of infection may also be different; preterm neonates may be more at risk of infection from iatrogenic sources or acquire vertically transmitted early-onset sepsis (50), whilst pediatric patients may be more likely to develop sepsis from a hospital-acquired infection such as methicillin-resistant *Staphylococcus aureus* if they have a chronic illness, or community-acquired meningococcal infection if they are otherwise healthy (3). Accordingly therapeutic considerations will differ (3), and it is not inevitable that IgM-enriched immunoglobulin will have an equal benefit in both populations. In the pediatric analysis, the observed significant benefit following adjunctive treatment with IgM-enriched immunoglobulin was based on just two studies, highlighting the need for further larger studies to validate these results.

Due to the fundamental differences in the neonatal and pediatric immune systems and the small number of pediatric studies, subgroup analyses were limited to studies conducted in neonates. Overall, IgM-enriched immunoglobulin treatment reduced the mortality risk in neonates by 55% compared with controls. Of the 13 neonatal studies included in the analysis, only two studies observed no effect of IgM-enriched immunoglobulin treatment on mortality risk (44, 46). Boonsopa et al. reported no deaths for either control or the IgM-enriched immunoglobulin treatment group (44), but noted improvements in respiratory rates, mean arterial pressure and serum pH with IgM-enriched immunoglobulin treatment, suggesting a clinical benefit. Akdag et al, reported similar mortality rates in neonates treated with IgM-enriched immunoglobulin compared with controls (46). However the authors also analyzed pro-inflammatory biomarkers including cluster of differentiation 64 (CD64) which has high sensitivity and specificity in children as a diagnostic marker of infection and sepsis (53). They observed a significant reduction in CD64 levels with IgM-enriched immunoglobulin treatment compared with controls and concluded that IgM-enriched immunoglobulin may support a reduction of infection.

Interestingly, the comparative risk of mortality between IgM-enriched immunoglobulin-treated and control patients was influenced by the design of the study, with a significant reduction (*p* = 0.04) in risk of mortality observed with prospective vs. retrospective studies. In our analysis, the secondary endpoints from both Capasso et al. retrospective studies (41, 42) were selected to be more analogous with studies where mortality was evaluated at discharge (43, 45–47). However, despite no reduction in total mortality with IgM-enriched immunoglobulin therapy (41, 42), Capasso et al. still observed a >20% reduction in short-term mortality in both analyses. RCTs are considered to have a lower risk of bias and confounding variables when compared with retrospective analyses. The significant effect observed in the prospective studies in this analysis adds credence to the overall beneficial effect of adjunctive therapy with IgM-enriched immunoglobulin in neonates with sepsis.

Adjunctive therapy with IgM-enriched immunoglobulin in patients with proven sepsis saw a greater reduction in risk of mortality when compared with patients with suspected sepsis. In the Ohlsson Cochrane Database systematic review (30), the authors commented that they had excluded comparisons where sepsis was subsequently proven, as a diagnosis of sepsis is often not confirmed at the time of treatment initiation. Whilst this is an important perspective, three more recent studies included in this analysis waited for confirmation of sepsis by either positive blood or cerebrospinal fluid culture before initiating adjunctive therapy with IgM-enriched immunoglobulin (36, 43, 49), and still saw a significant mortality benefit compared with controls. Nevertheless, early administration of IgM-enriched immunoglobulin has been shown to be an important consideration and early treatment has been associated with a reduced risk of in-ICU mortality in adult patients with septic shock (54).

The INIS study, an RCT with more than 3,000 neonates, evaluated the administration of normal human low-dose IVIG (500 mg/kg; two doses, 48 h apart) in neonatal sepsis and concluded that there was no mortality benefit with standard IVIG adjunctive therapy (18). It was established that for treatment of neonatal sepsis, benefits of immunoglobulin are dependent on the type of immunoglobulin solution and on dose (18). Previous systematic reviews have performed subgroup analysis on IgM-enriched immunoglobulin therapy for neonatal sepsis (29–31). Whilst Kreymann et al. (31) observed a 50% reduction in risk of mortality compared with controls, Alejandria et al. (29) concluded that the evidence was not sufficient to draw robust conclusions and Ohlsson et al. (30) concluded that IgM-enriched immunoglobulin therapy was unable to reduce the risk of mortality. Since these meta-analyses, a further six studies on IgM-enriched immunoglobulin adjunctive therapy in neonatal sepsis have been published (36, 42–44, 46, 49). In total, these studies included 1,036 patients, of which three were prospective studies (36, 43, 49) and three retrospective analyses (42, 44, 46).

The retrospective PIGMENT study, which included 254 pediatric patients with a median age of 13 months with either sepsis, septic shock or multi-organ failure, compared 3-day and 5-day treatment with IgM-enriched immunoglobulin adjunctive therapy (250 mg/kg/day) (55). Although the absence of a control group precluded its inclusion in the current analysis, the study reported a significant reduction in mortality rate with longer treatment durations. In our study, a longer treatment duration was also associated with significantly reduced mortality compared with the SmPC-recommended 3-day regimen (35).

More contemporary studies are often more rigorous and consider advances in standard of care for sepsis and advances in antibiotic therapies. Interestingly, no difference was observed between studies conducted pre- and post- 2000 which may reflect the increases in antibiotic resistance rates now seen in hospitals.

The relationship between immunoglobulin levels at diagnosis of sepsis or septic shock and the outcome is controversial. Studies which evaluate the impact of IgG levels on mortality risk in adult patients with sepsis have reported conflicting outcomes (56–62), and similar disparities have been reported on the association of IgA levels and mortality risk (54, 57, 61). Whilst the data for IgM appear to be marginally more prognostic, with several studies in adults reporting a significant association between decreased IgM levels and reduced survival, at sepsis onset (63) and over time (54, 57, 64), two studies reported no association between IgM levels and survival (56, 61). Notably, Bermejo et al. evaluated the synergy between the three immunoglobulin isotypes and observed that the greatest risk of mortality was associated with the combined deficiency of IgA, IgG and IgM (57).

In view of these observations, adjunctive IgM-enriched immunoglobulin preparations with increased concentrations of IgM and IgA (12% each) may be more effective than standard human IVIG preparations. Berlot et al. evaluated treatment with IgM-enriched immunoglobulin in adult patients with sepsis and observed significant increases in levels of IgM and IgA (but not IgG) over time in survivors compared with non-survivors (65). In addition, Haque et al. also observed significant increases in serum IgM and IgA levels (but not IgG) in neonates treated with IgM-enriched immunoglobulin compared with controls (37). This suggests that IgM-enriched immunoglobulin adjunctive therapy may have the potential to supplement a patient's humoral immunity to within normal parameters.

IgM mediates a range of immune defenses; it is a potent activator of the complement system and is required for the maximal induction of the IgG antibody response (66). IgM also rapidly removes self-antigens, such as apoptotic cell debris, preventing the stimulation of further inflammatory responses (66). As a result, IgM has higher opsonization activity and activation of complement compared with IgG, and accordingly IgM-enriched immunoglobulin have been shown to have higher antimicrobial activity than immunoglobulin preparations containing IgG alone (19, 20, 67, 68). IgA is the second most prevalent antibody and induces either anti-pathogenic or immunomodulatory effects, especially on neutrophils. Therefore, in addition to IgM, the IgA component of the IgM-enriched immunoglobulin preparation could mediate beneficial effects compared with standard IVIG (69).

This systematic review has several limitations. Firstly study characteristics were varied with different IgM-enriched immunoglobulin treatment durations, different control arms and/or study designs and importantly an inconsistent definition of mortality. Additionally for neonates there were differences in undefined early- vs. late-onset sepsis, gestational age and birthweight, all of which will have a significant impact on the neonate's susceptibility to sepsis. Due to the limited number of studies available and the disparities in study reporting, the effect of these parameters on mortality benefit cannot be evaluated in this analysis. The complex nature of sepsis itself makes it very difficult to ascertain the cause of death in neonates and pediatric patients; causes of mortality in sepsis are multifactorial and treatment is one of many factors that may influence mortality risk in these patients. Finally, given the 34-year timespan over which the studies included in the analyses were conducted, there will have been many advances in intensive care medicine. This however, has been tempered by new challenges associated with increases in antimicrobial-resistance (70).

## 5. Conclusions

This meta-analysis and systematic review have shown that IgM-enriched immunoglobulin adjunctive therapy may reduce the risk of mortality in neonatal and pediatric patients with sepsis when compared with controls. Whilst there are limitations with the studies included in this analysis, this systematic review reveals a clear mortality benefit when prospective studies only were evaluated, highlighting the need for additional large multicentered RCTs to substantiate and further evaluate the observations of our analysis.

## Data Availability

The raw data supporting the conclusions of this article will be made available by the authors, without undue reservation.
