# Supplementary material for: Clinical efficacy of IgM-enriched immunoglobulin as adjunctive therapy in neonatal and pediatric sepsis: a systematic review and meta-analysis
Source: Front Pediatr. 2023 Aug 11;11:1239014. doi: 10.3389/fped.2023.1239014 (PMC10451087; doi:10.3389/fped.2023.1239014)
Supplement: Supplementary file 1 [file Table1.docx]

**Supplementary Materials**

Clinical efficacy of IgM-enriched immunoglobulin as adjunctive therapy in neonatal and pediatric sepsis: a systematic review and meta-analysis

Ener Cagri Dinleyici ^1*^, Georg Frey ^2^, Ermira Kola^3^, Ulrike Wippermann^4^, Artur Bauhofer^4^, Alexander Staus^4^, Peter Griffiths^5^, Muhamad Azharry^6^, Rinawati Rohsiswatmo^6^.

^1^Eskisehir Osmangazi University Faculty of Medicine, Department of Pediatrics, Eskisehir Turkiy;  ^2^Darmstädter Kinderkliniken Prinzessin Margaret; Klinik für Neonatologie, Perinatalzentrum Südhessen, Darmstadt, Germany; ^3^University Hospital Center "Mother Teresa"; Pediatrics Intensive Care Unit; Tirana AL, Albania; ^4^Biotest AG, Dreieich, Germany; ^5^Biotest UK, Birmingham, UK; ^6^Cipto Mangunkusumo National Central General Hospital, Jakarta, Indonesia.

***Correspondence:**Ener Cagri Dinleyici
timboothtr@yahoo.com

##
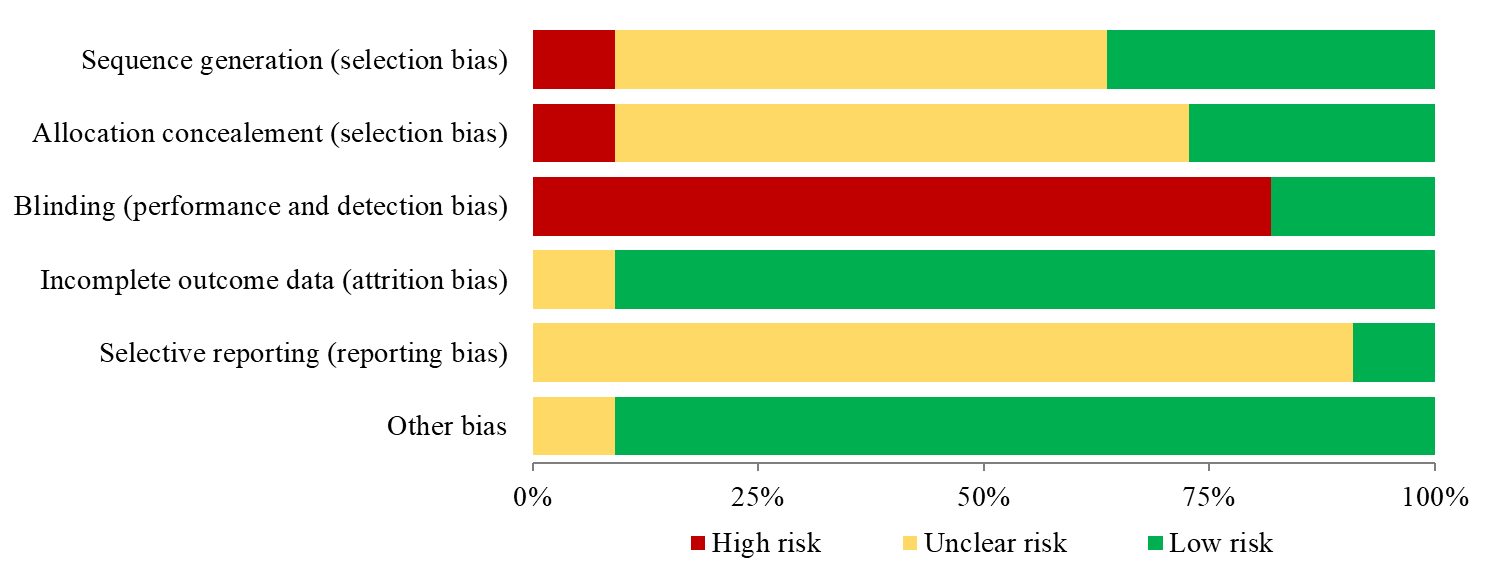


## Figure S1 Cochrane Collaboration’s risk of bias graph: review authors’ judgment about the risk of bias across each criteria for all randomised controlled studies.


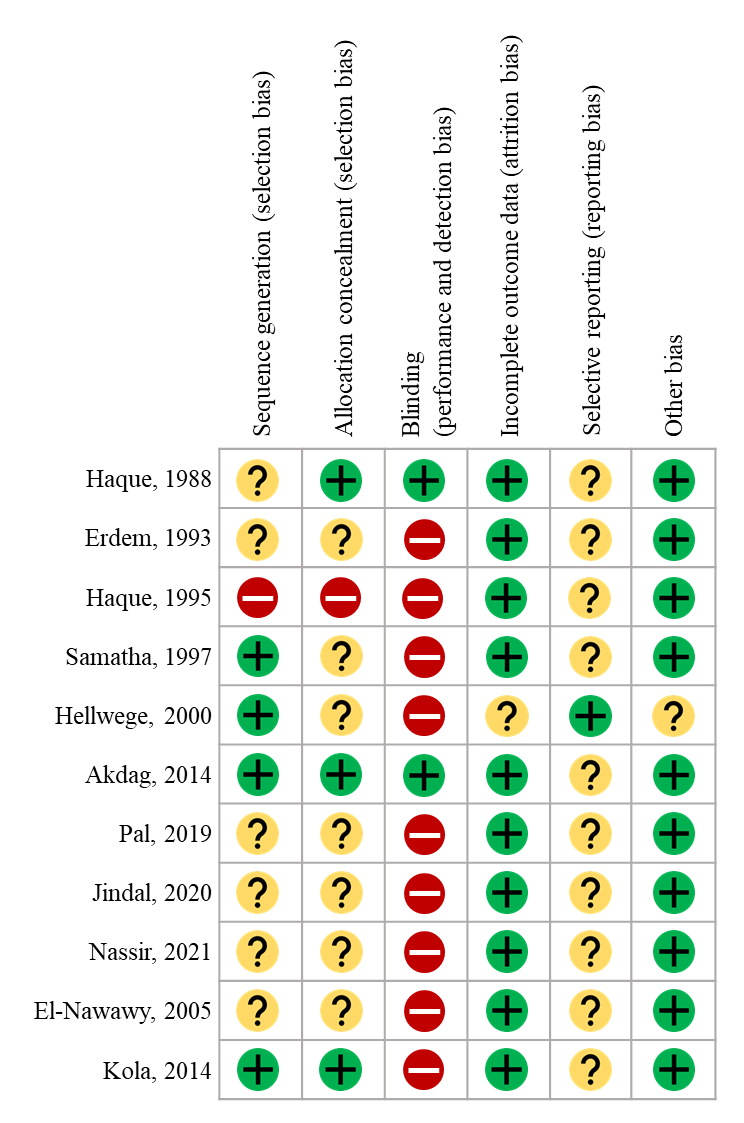


## Figure S2 Cochrane Collaboration’s risk of bias summary: review author’s judgments about each risk of bias criteria for each included RCT.


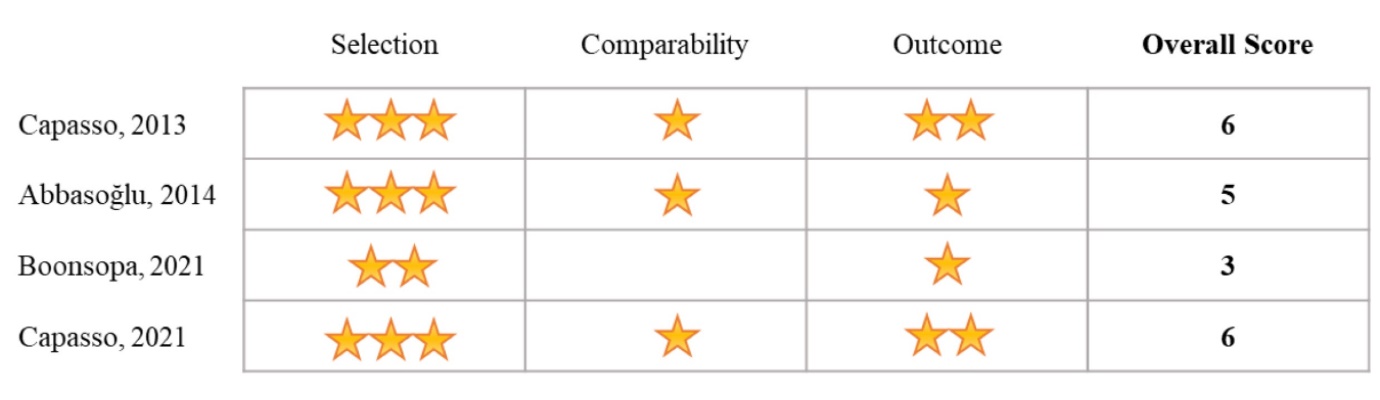


## Figure S3 Newcastle–Ottawa Scale for quality analysis: review author’s judgments about the methodological quality of included non-randomized studies (high-quality studies ≥6 stars).
